# Henoch-Schönlein Purpura Presenting in Association With Neuroblastoma: A Case Report

**DOI:** 10.3389/fped.2020.00077

**Published:** 2020-02-28

**Authors:** Zineb Alfath, Asmaa Ferdjallah, Emily Greengard, Sabeen K. Askari, Karim T. Sadak, Colleen K. Correll

**Affiliations:** ^1^University of Minnesota Medical Center, Minneapolis, MN, United States; ^2^Division of Pediatric Hematology & Oncology, University of Minnesota, Minneapolis, MN, United States; ^3^MHealth Fairview Pathology Service Line, Minneapolis, MN, United States; ^4^Division of Pediatric Rheumatology, University of Minnesota, Minneapolis, MN, United States

**Keywords:** neuroblastoma, Henoch-Schönlein purpura, rash, pediatrics, case report

## Abstract

Henoch-Schönlein purpura (HSP) is a common systemic vasculitis affecting children. It is managed in the outpatient setting and rarely associated with malignancy. We present a case of neuroblastoma in a 7-year-old boy diagnosed after suspected HSP. Our case highlights the importance of maintaining a broad differential diagnosis in children with atypical HSP and performing a skin biopsy with immunofluorescence when a rash is present.

## Introduction

Henoch-Schönlein purpura (HSP) is the most common vasculitis affecting children (1). It is an immunoglobulin (Ig)A-mediated leukocytoclastic vasculitis in which IgA-containing immune complexes deposit in the small vessels of the skin, gastrointestinal tract, and kidneys. HSP is more common in children than adults and although the cause is not clear, it has been associated with preceding infection—especially group A streptococcal infections, staphylococcal infections, and parainfluenza (2). Several solid tumors and hematologic malignancies have been reported to induce or be associated with adult-onset HSP, but the association is rare and poorly understood (3–7). Malignancy is even less commonly associated with childhood-onset HSP (8–10). Here we present a case of a young boy who presented with clinical features of HSP who was ultimately diagnosed with neuroblastoma.

## Case Report

A 7-year-old boy with a history of a port wine stain and dysfunctional elimination syndrome presented to his local medical center with a 3-day history of an erythematous, non-blanching rash on the legs, arthralgias involving the lower extremities, and pain in his abdomen, groin, and scrotum. At this visit, it was noted that he had a temperature of 100.4°F, face, penis, and scrotum swelling, and scattered, non-blanching purpuric lesions on the lower legs and groin. Urinalysis done at this visit showed positive protein (not quantified), 0–5 white blood cells (WBC), and 0–2 red blood cells (RBC). Other lab results were normal as follows: creatinine (Cr) 0.42 mg/dl, WBC 7.0 10 e9/L with a normal differential, hemoglobin (hgb) 10.6 g/dl, mean corpuscular volume (MCV) 82 fl, and platelets (plts) 249 10 e9/L. Based upon these clinical findings, he was diagnosed with HSP and instructed to follow up with his primary care provider (PCP). Supportive measures and reassurance were provided. Two weeks later, he continued to experience worsening arthralgias and reported intermittent fevers, but he had some improvement in his rash. The PCP prescribed a 3-week tapered dose of prednisolone starting at 10 mg per day, and he began showing signs of improvement. However, during his third week of treatment with prednisolone, he again presented to the emergency department with new and worsening symptoms including progressive fatigue, persistent fever with temperature up to 102°F, and intermittent joint pain, with episodes of vomiting and epistaxis. Repeat labs showed a mild normocytic anemia (hgb 10 g/dL) and elevated C-reactive protein (CRP) of 7.33 mg/dL (normal < 0.5 mg/dL).

**Figure 1:**
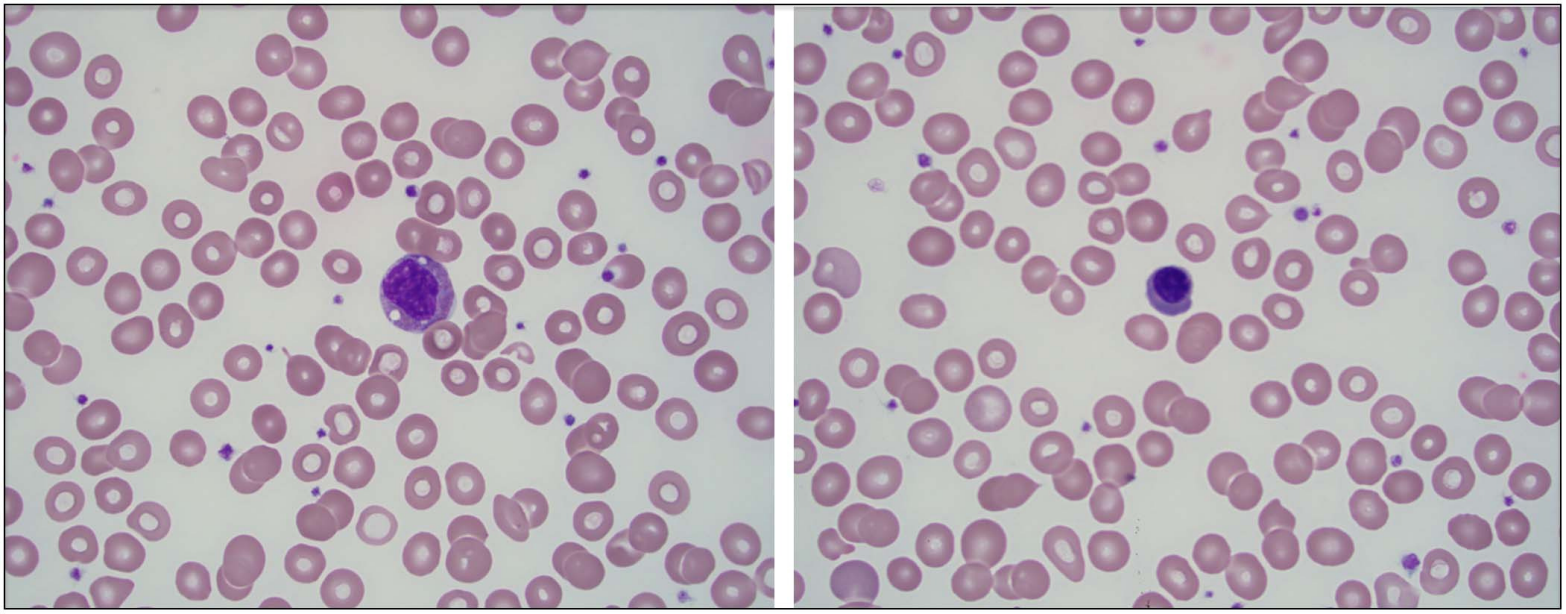
Mild leukoerythroblastic reaction seen on peripheral blood smear with slightly left-shifted granulocyte series and rare circulating nucleated red blood cells. **(A)** A granulocyte precursor is seen at the center of the field (100×). **(B)** A nucleated red blood cell is seen at the center of the field (100×).

A few days later, ~2 months after the initial onset of his symptoms, the patient was evaluated by pediatric rheumatology for his ongoing symptoms. He had recently completed the 3-week tapered course of prednisolone. His rash and abdominal pain had resolved. On exam, he appeared significantly fatigued, grimaced with range of motion of the neck and shoulders, and cringed when his hips were abducted. No joint effusions were noted. A diagnosis of recalcitrant HSP was considered, along with the possibility of another systemic vasculitis, inflammatory bowel disease, systemic juvenile idiopathic arthritis, macrophage activation syndrome, and malignancy. Further labs were obtained and results included the following: CRP 7.1 mg/dL (normal < 0.5 mg/dL), erythrocyte sedimentation rate (ESR) 78 mm/h, elevated lactate dehydrogenase (LDH) (345 U/L), worsening microcytic anemia (hgb 8.7 g/dl), hypoalbuminemia (3.0 g/dL), a normal uric acid (2.2 mg/dL), and an overall normal urinalysis (UA) without hematuria but with protein 10 mg/dl, 6 WBC, and 1 RBC. A peripheral blood smear, performed to rule out an oncologic disorder, demonstrated a mild leukoerythroblastic reaction, suggesting a bone marrow infiltration process (Figure 1). The patient was subsequently referred to pediatric oncology for further evaluation.

**Figure 2:**
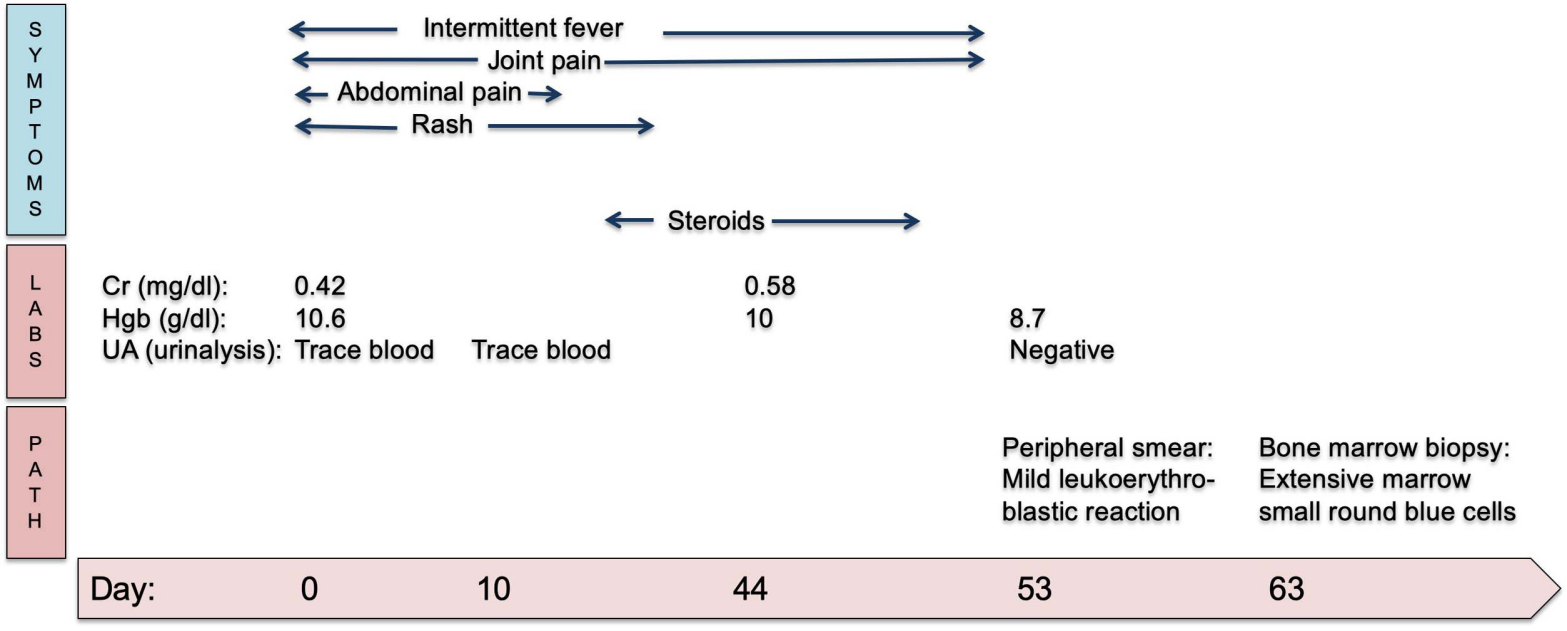
Timeline in days of clinical symptoms of HSP and eventual progression to neuroblastoma diagnosis. Cr, creatinine; Hgb, hemoglobin; UA, urinalysis.

A unilateral bone marrow biopsy showed extensive involvement by a non-hematopoietic malignancy with features suggestive of neuroblastoma. Follow-up computerized tomography (CT) imaging revealed a heterogeneous, partially calcified tumor in the right hepatorenal recess with widespread metastatic disease in the axial spine without spinal canal infiltration. A biopsy of the primary tumor identified poorly differentiated neuroblastoma, n-myc non-amplified. Urine catecholamines were elevated. A metaiodobenzylguanidine (MIBG) scan demonstrated diffuse uptake in the primary tumor and diffuse osseous metastatic disease. Given the presence of metastatic disease, he was classified as having International Neuroblastoma Risk Group (INRG) Stage M disease (International Neuroblastoma Staging System Stage 4). He completed five cycles of chemotherapy followed by tandem autologous hematopoietic stem cell transplantation, radiation and immunotherapy as per Children's Oncology Group (COG) standard of care for high-risk neuroblastoma therapy (11). His end of therapy evaluations revealed no evidence of disease and he remains in remission. A summary of his clinical course can be seen in Figure 2.

## Discussion

This case report (see Appendix) highlights an example of a patient with HSP who was later diagnosed with neuroblastoma. The phenomenon of HSP associated with malignancy has been described in the literature but typically in older males with a median age of 60 (6, 12). This patient presented with classic features of HSP including lower extremity purpuric rash, abdominal pain and arthralgias, and scrotal swelling (1). However, he also had atypical features that prompted further evaluation for an underlying disease. These atypical features included recurrent fever, severe limb pain out of proportion to that typically seen in HSP and without arthritis, a high degree of systemic inflammation, and long length of course.

HSP is the most common type of vasculitis in childhood and is often managed by general pediatricians (13). HSP is diagnosed based upon clinical features of palpable purpura or petechiae with lower limb predominance and at least one of the following: abdominal pain, typical skin or renal histopathology, arthritis or arthralgia, and renal involvement (14). Although this classification criteria is highly sensitive (100%) and specific (87%), it is important for providers to maintain a differential diagnosis in patients presenting with these features (14). Our patient met diagnostic criteria with purpuric rash, abdominal pain, and arthralgia. Additionally, he presented with transient scrotal swelling, a recognized manifestation of HSP although not pathognomonic (14). The combination of severe limb pain, fevers, and systemic inflammation in a child should prompt consideration of hematologic malignancy (15). Neuroblastoma is a tumor of primitive neuroblasts of the embryonic neural crest, occurring anywhere along the sympathetic nervous system with a primary site often in the adrenal medulla. Rare cases of metastatic cutaneous lesions of neuroblastoma are noted in the literature and are typically associated with neonatal or infantile neuroblastoma (16). Symptoms of neuroblastoma may include abdominal pain, vomiting, constipation, nausea, and bone pain among others. In this patient's case, his skin eruption, abdominal pain and arthralgia may have been solely caused by the neuroblastoma.

HSP is not traditionally felt to be a paraneoplastic syndrome of neuroblastoma and to our knowledge there is only a single published case report describing an incidental finding of neuroblastoma on CT scan for abdominal pain in a 5-year-old child diagnosed and treated for HSP (10). While both this patient and our patient presented similarly, our patient's rash resolved prior to evaluation by pediatric oncology and diagnosis of neuroblastoma. Our patient also had additional concerning symptoms such as intermittent fever and limb pain that were felt to be atypical for HSP.

Although the mechanism is not completely understood, there is concern that neoplastic antigens could lead to the formation of immune complexes, which induce the lesions of HSP (3). Podjasek et al. noted, in a patient cohort of 15 individuals with cutaneous small-vessel vasculitis secondary to solid-organ malignancy, the absence of papillary dermal inflammation and edema, lymphocytes and plasma cells on histopathology was found to be a statistically significant finding (4). This patient did not have a skin biopsy at the time of diagnosis of HSP, however, the features of this patient's rash, along with these other clinical features, were concerning for HSP. Nevertheless, a skin biopsy could have potentially distinguished a classic HSP, IgA-mediated leukocytoclastic vasculitis from another malignancy-induced small vessel vasculitis.

The association between malignancy and vasculitis in this patient remains unclear—the HSP may have developed independently, as a paraneoplastic syndrome, or as a manifestation of his metastatic disease. A limitation of this case, however, is that the authors of this case did not see the rash firsthand. By the time the patient presented to the pediatric rheumatology clinic, the rash had resolved. The classification criteria for HSP requires that a patient have palpable purpura or petechiae predominant in the lower extremities. We trust that this patient's local provider accurately assessed the rash as non-blanching and purpuric. In this case, the purpuric rash was suggestive of HSP. Unfortunately, a photo was not available for inclusion in this case report.

Although HSP is generally self-limited and managed in the primary care setting, patients who meet the diagnostic criteria for HSP should still be assessed closely for atypical findings such as severe joint pain or limb pain, persistent fevers, and significantly elevated inflammatory markers. Additionally, early universal treatment with corticosteroids has not been shown to prevent renal or gastrointestinal complications of HSP and thus should be weighed against the potential to mask signs of acute infection or malignancy (17). This case highlights the importance of maintaining a broad differential in the setting of recalcitrant HSP. Providers should utilize laboratory markers and peripheral smear findings to guide the diagnostic work-up, and exercise caution when prescribing steroids for atypical presentations of vasculitis.

## Data Availability Statement

The raw data supporting the conclusions of this article will be made available by the authors, without undue reservation, to any qualified researcher.

## Ethics Statement

Written informed consent was obtained from the parents for publication of this case report.

## Author Contributions

ZA, AF, EG, KS, and CC conceptualized and designed the study, drafted the initial manuscript, and reviewed and revised the manuscript. All authors approved the final manuscript as submitted and agree to be accountable for all aspects of the work.

### Conflict of Interest

The authors declare that the research was conducted in the absence of any commercial or financial relationships that could be construed as a potential conflict of interest.

## Abbreviations

COG: Children's Oncology Group
Cr: creatinine
F: Fahrenheit
WBC: white blood cell
N: neutrophil
L: lymphocyte
M: monocyte
E: eosinophil
Hgb: hemoglobin
MCV: mean corpuscular volume
Plts: platelets
INR: international normalized ratio
CRP: C-reactive protein
ESR: erythrocyte sedimentation rate
LDH: lactate dehydrogenase
MIBG: metaiodobenzylguanidine
PCP: primary care provider
UA: urinalysis.

## Appendix

**Table:**

**CARE Guidelines for Reporting Case Reports**	
Title—The diagnosis or intervention of primary focus followed by the words “case report”	✓
Key Words—2 to 5 key words that identify diagnoses or interventions in this case report (including “case report”)	✓
Abstract—Introduction- What is unique about this case and what does it add to the scientific literature? The patient's main concerns and important clinical findings. The primary diagnoses, interventions, and outcomes. Conclusion—What are one or more “take-away” lessons from this case report?	✓
Introduction—Briefly summarizes why this case is unique and may include medical literature references. Patient information—De-identified patient specific information, primary concerns and symptoms of the patient, Medical, family, and psychological history including relevant genetic information, Relevant past interventions and their outcomes	✓
Clinical findings—Describe significant physical examination (PE) and important clinical findings. Timeline—Historical and current information from this episode of care organized as a timeline (figure or table)	✓ ✓
Diagnostic assessment—Diagnostic methods (PE, laboratory testing, imaging, surveys), Diagnostic challenges, Diagnosis (including other diagnoses considered), Prognostic characteristics when applicable	✓
Therapeutic intervention—Types of therapeutic intervention (pharmacologic, surgical, preventative), Administration of therapeutic intervention (dosage, strength, duration), Changes in therapeutic interventions with explanations	✓
Follow-up and Outcomes—Clinician- and patient-assessed outcomes if available, Important follow-up diagnostic and other test results, Intervention adherence and tolerability (How was this assessed?), Adverse and unanticipated events	✓
Discussion—Strengths and limitations in your approach to this case, Discussion of the relevant medical literature, The rationale for your conclusions, The primary “take-away” lessons from this case report (without references_ in a one paragraph conclusion)	✓
Patient Perspective—The patient should share their perspective on the treatment(s) they received	Not applicable for our patient as he is a pediatric patient.
Informed Consent—The patient should give informed consent (Provide if requested).	✓
